# A Retrospective Analysis of the Effect of Irinotecan-Based Regimens in Patients With Metastatic Breast Cancer Previously Treated With Anthracyclines and Taxanes

**DOI:** 10.3389/fonc.2021.654974

**Published:** 2021-11-22

**Authors:** Jiaojiao Suo, Xiaorong Zhong, Ping He, Hong Zheng, Tinglun Tian, Xi Yan, Ting Luo

**Affiliations:** ^1^ Department of Head, Neck and Mammary Gland Oncology, Cancer Center, West China Hospital, Sichuan University, Chengdu, China; ^2^ Laboratory of Molecular Diagnosis of Cancer, Clinical Research Center for Breast, West China Hospital, Sichuan University, Chengdu, China

**Keywords:** irinotecan, metastatic breast cancer, efficacy, chemotherapy, palliative therapy

## Abstract

**Background:**

At present, patients with metastatic breast cancer (MBC) have few treatment options after receiving anthracyclines and taxanes. Studies have shown that irinotecan has modest systemic activity in some patients previously treated with anthracyclines and taxanes. This study aimed to evaluate the efficacy of irinotecan-based chemotherapy for breast cancer patients in a metastatic setting.

**Methods:**

We retrospectively collected the clinical information and survival data of 51 patients with MBC who received irinotecan at West China Hospital of Sichuan University. The primary endpoints were the progression free survival (PFS) and overall survival (OS), and the secondary endpoint was the objective response rate (ORR). To minimize potential confounding factors, we matched 51 patients who received third-line chemotherapy without irinotecan through propensity score matching (PSM) based on age, hormone receptor (HR), and human epidermal growth factor receptor 2 (HER2), compared their OS and PFS rates to those treated with irinotecan.

**Results:**

From July 2012 to October 2020, 51 patients were treated with an irinotecan-containing regimen. The median number of previous treatment lines was 4, and a median of two previous chemotherapy cycles (ranging from 1–14 cycles) were given in a salvage line setting. The ORR was 15.7%, and the disease control rate (DCR) was 37.3%. For the irinotecan group, the median PFS was 3.2 months (95% CI 2.7–3.7), while the median OS was 33.1 months (95% CI 27.9–38.3). Univariate analysis results suggested that irinotecan could improve PFS in patients with visceral metastasis (P=0.031), which was 0.7 months longer than patients without visceral metastasis (3.5 months *vs.* 2.8 months). Compared to the patients who received third-line non-irinotecan chemotherapy, the irinotecan group showed a longer trend of PFS without statistical significance (3.2 months *vs* 2.1 months, P = 0.052). Similarly, the OS of the irinotecan group was longer than the third-line survival without irinotecan, but it was not statistically significant (33.1 months *vs* 18.0 months, P = 0.072).

**Conclusions:**

For MBC patients who were previously treated with anthracyclines and/or taxanes, an irinotecan-containing regimen achieved moderate objective response and showed a trend of survival benefit, which deserves further study.

## Introduction

Globally, breast cancer is the most common cancer and the leading cause of cancer death in women (1). The incidence of breast cancer has been rising, and this trend is expected to continue. Long-term survival mainly depends on tumor stage and molecular subtype. Early detection and early treatment are important strategies for improving prognosis. The 5-year survival rate of those diagnosed with early breast cancer is 99%, while that of those diagnosed with metastatic breast cancer (MBC) is 25% (2, 3). In the past few decades, significant progress has been made in improving the survival rate of patients with MBC, but most cannot be cured by existing treatment methods (4, 5). In patients with rapid tumor progression or life-threatening visceral metastasis, or those who need to quickly control tumor progression or relieve symptoms, combination chemotherapy is usually appropriate (6).There is currently no standard chemotherapy regimen for MBC (7). The available treatment options include anthracyclines, taxanes, 5-fluorouracil, vinorelbine, gemcitabine (5, 8). Those breast cancer patients with relatively long survival time often face the dilemma that no effective drugs are available. Irinotecan is a topoisomerase I inhibitor, which is widely used in clinical treatment of advanced colorectal cancer, lung cancer, gastric cancer (9–11). A few clinical trials have shown that irinotecan had modest systemic activity in some patients previously treated with anthracyclines and taxanes. The objective response rate (ORR) of patients with MBC who received irinotecan monotherapy was 5%–23%, while the ORR of patients with MBC who received a combination of irinotecan and various chemotherapy drugs ranged from 14%–64%, usually including patients who had been heavily pretreated (5, 12, 13). Irinotecan has not been regarded as a routine treatment option for patients with MBC, and the outcome of subsequent therapy with irinotecan in patients with MBC was not clear. The aim of the present study was to evaluate the efficacy of irinotecan as a salvage line therapy for patients with MBC.

## Patients and Methods

We enrolled patients with MBC who were admitted to West China Hospital from July 1, 2012 to October 30, 2020 and were registered in the Breast Cancer Information Management System (BCIMS). The BCIMS prospectively records patient clinical and pathological characteristics, medical history, diagnoses, laboratory results, treatments, and follow-up data (14).

Eligibility criteria included (1) Patients with MBC, that is pathologically diagnosed breast cancer with metastasis sites, including skin, lymph node (non-breast lymphatic drainage area),bone and other visceral metastasis and (2) Patients received systemic chemotherapy with or without irinotecan in a salvage line. The prior treatment regimens and lines for metastatic disease were not limited. Of the 1607 patients in the database, fifty-one patients treated with irinotecan met the inclusion criteria of the irinotecan group. Patients with no irinotecan medication record (1556 cases) in the database were matched through propensity score matching (PSM) in a 1:1 ratio as the control group, and the matching factor was age ( ± 5years), hormone receptor (HR), human epidermal growth factor receptor 2 (HER2) and the number of treatment lines was three lines or above. Then, two matched cohorts of 51 patients were created. Their data, including basic information, diagnosis, molecular subtypes, chemotherapy regimens, evaluation of efficacy, were exported from BCIMS.

### Therapeutic Schedule

Patients were treated with intravenous irinotecan 125 mg/m^2^ weekly for 4 weeks followed by a 2-week break. This regime was based on one randomized Phase II trial with irinotecan in MBC, which showed that weekly treatment schedules, compared with every 3 weeks, had better response rates (15). Irinotecan was combined with a variety of other chemotherapeutics, including 5-FU analogs, vinorelbine, gemcitabine, and platinum, as well as being combined with various biologic agents, such as trastuzumab, apatinib (**Table 1**).

**Table 1:** Summary of treatment options in this study.

Treatment regimen	N = 51, %
Irinotecan monotherapy	4 (7.8)
Irinotecan+ anti-angiogenesis+/-target therapy	3 (5.9)
Irinotecan +5-FU analogs+/-anti-angiogenesis	33 (64.7)
Irinotecan +platinum+/-anti-angiogenesis	5 (9.8)
Irinotecan + vinorelbine +/- anti-angiogenesis	3 (5.9)
Irinotecan + gemcitabine	2 (3.9)
Irinotecan + docetaxel	1 (2.0)

### Efficacy Evaluation

The ORR was defined as the objective response rate—that is, the ratio of patients with complete response (CR) plus partial response (PR) to all patients. The disease control rate (DCR) was defined as the ratio of CR+PR+SD (stable disease) patients to all patients. Progression free survival (PFS) was defined as the time from initiation of irinotecan to the presence of objective evidence of disease progression (or death for any reason). Overall survival (OS) was defined as the time from initiation of irinotecan until death, or loss of follow-up or reaching the study observation deadline. Follow-up was conducted *via* telephone or medical visit until death. Lost to follow-up was defined as failure to make contact with the patient on > 2 consecutive occasions (16). The longest follow-up time was 40 months. According to response evaluation criteria in solid tumors 1.1 (RECIST 1.1), the therapeutic effect should be evaluated through imaging examination about 2 cycles. The primary endpoints were PFS and OS, and the secondary endpoint was ORR. 

### Analysis Methods

Survival analysis was performed using SPSS version 25.0. A survival curve was created using the Kaplan–Meier method. A log-rank test was used for univariate analysis of PFS and OS. Categorical variables were compared with the χ2 test or Fisher’s exact test. PSM was conducted using R software (version 4.0.3), employing a 1:1 nearest neighbor with a caliper of 0.02. Subgroup analysis was performed with R software (version 4.0.3). P < 0.05 was considered statistically significant.

## Results

### Baseline Characteristics of Subjects

A total of 51 patients with MBC entered the irinotecan group and the control group, respectively. The characteristics of the two groups were roughly similar. Almost all patients in both groups were female. The median patient age was 43 years, and premenopausal patients accounted for more than 60% of the patients. The biological subtype included estrogen receptor (ER)-positive and/or progesterone receptor (PR)-positive (74.5%), HER2-positive (35.3%), and triple negative (13.7%). Visceral metastasis had occurred in more than 85% of the patients, more than 85% of the patients had previously received anthracyclines, and more than 95% had been treated with taxanes. The median number of previous treatment lines was 4, and a median of two previous chemotherapy cycles (ranging from 1–14 cycles) were given in a salvage line setting. Demographic and clinical characteristics between the irinotecan and control groups are shown in **Table 2**.

**Table 2:** The baseline patient characteristics.

Characteristics	Irinotecan group (N, %)	Control group (N, %)	P value
Gender			1.000
female	50 (98.0)	51 (100)	
male	1 (2.0)	0 (0)	
Age, median (range, year)	43 (27-70)	43 (22-70)	0.839
<45	32 (62.7)	31 (60.8)	
≥45	19 (37.3)	20 (39.2)	
Menopausal status			0.532
Pre-menopause	32 (62.7)	35 (68.6)	
Post-menopause	19 (37.3)	16 (31.4)	
Biological subtype			0.873
HR(+)	38 (74.5)	38 (74.5)	
HER2(+)	18 (35.3)	17 (33.3)	
Triple negative	7 (13.7)	9 (17.6)	
Visceral metastasis			0.767
Yes	44 (86.3)	46 (88.2)	
No	7 (13.7)	5 (11.8)	
Prior anthracycline therapy			0.799
Yes	42 (82.4)	41 (80.4)	
No	9 (17.6)	10 (19.6)	
Prior taxane therapy			0.495
Yes	51 (100)	49 (96.1)	
No	0 (0)	2 (3.9)	

### Efficacy

**Figure 1:**
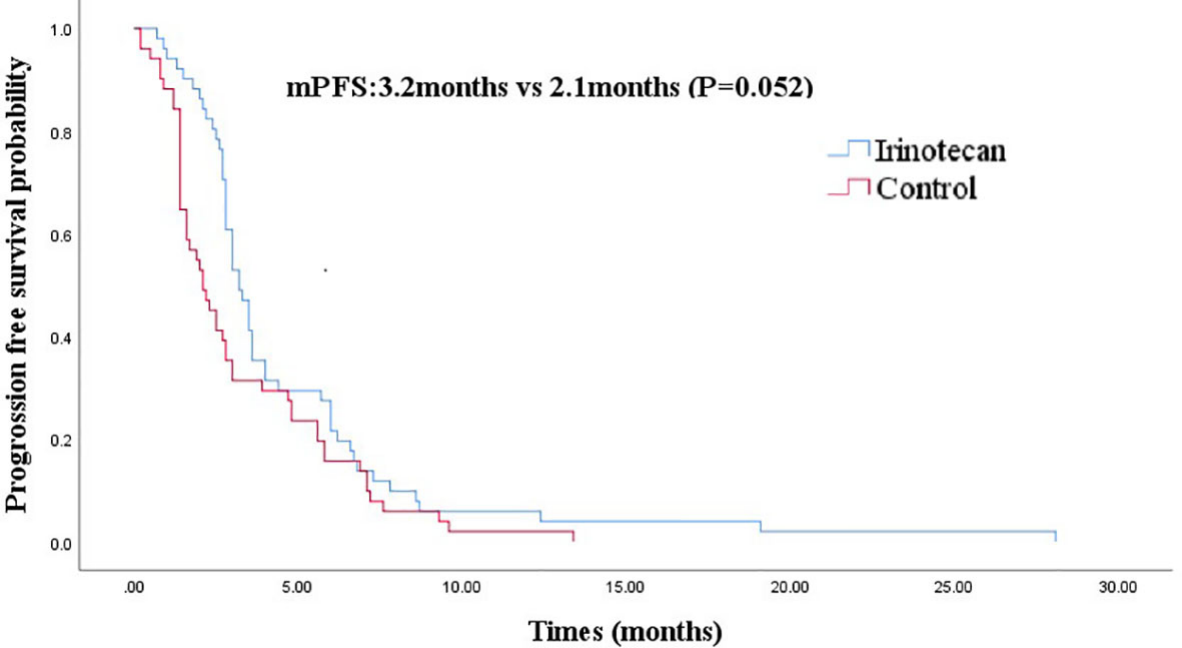
Comparison of progression-free survival in irinotecan group and third-line progression-free survival in control group.

**Figure 2:**
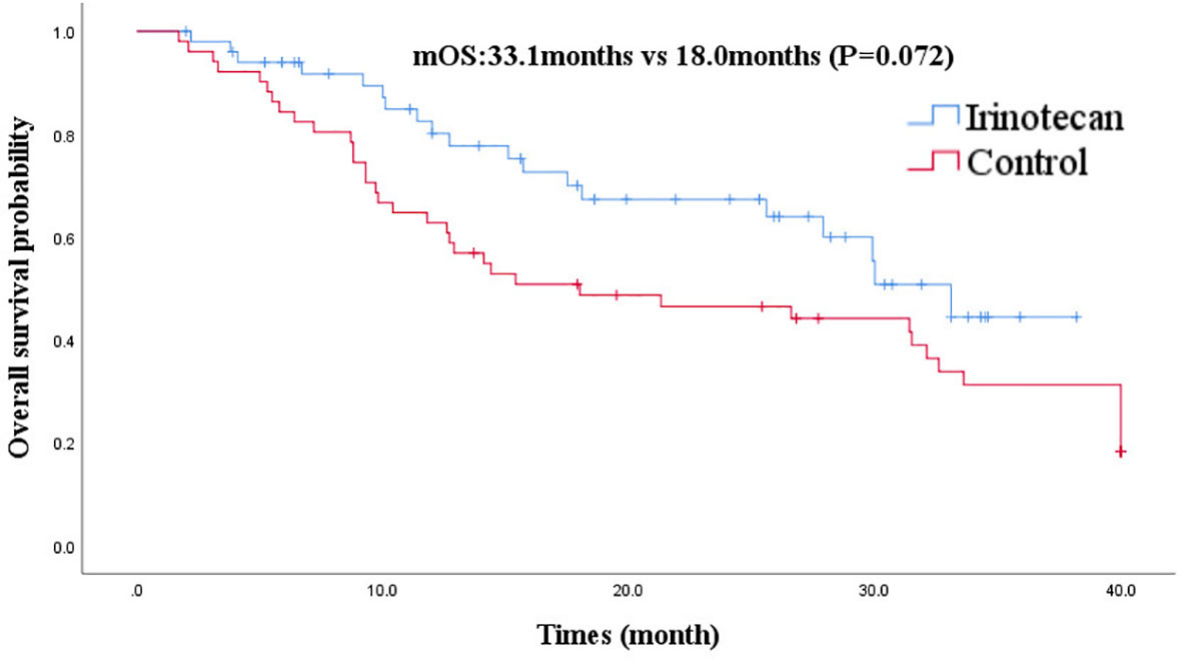
Comparison of overall survival in irinotecan group and third-line overall survival in control group.

At the cutoff of October 30, 2020, the best overall response of the irinotecan group was: CR (n =1), PR (n =7), SD (n = 11), progression disease (PD) (n = 32), an ORR of 15.7%, and a DCR of 37.3%. The median PFS for the irinotecan group was 3.2 months (95% CI 2.7–3.7) (**Figure 1**), the median OS was 33.1 months (95% CI 27.9–38.3), and the 2-year OS rate was 70.0% (**Figure 2**).

Results of the univariate analysis indicated that the PFS of the irinotecan group was significantly prolonged in patients with visceral metastasis (P = 0.031) compared with those without visceral metastases. Age (< 45 years, ≥ 45 years), menopausal status (pre-menopause, post-menopause), triple negative (positive/negative), HER2 status (positive/negative), HR status (positive/negative), and number of previous chemotherapy lines (≤ 3, >3) were not associated with the PFS of irinotecan (**Table 3**). With regard to OS, the univariate analysis found no clinicopathological factors affecting OS (**Table 3**).

**Table 3:** Univariate analysis of PFS and OS (Kaplan-Meier).

Variable	PFS	95% CI	P value	OS	95% CI	P value
Age			0.905			0.156
<45	3.0	2.2-3.8		30.0	16.7-43.3	
≥45	3.3	2.8-3.8		–	–	
Menopausal status			0.101			0.455
Pre-menopause	3.5	3.1-3.9		–	–	
Post-Menopause	2.8	2.7-2.9		27.9	12.2-43.6	
HR status			0.064			0.382
HR (+)	3.5	3.0-4.0		30.0	24.6-35.5	
HR (-)	2.8	2.6-3.0		–	–	
Triple negative			0.066			0.648
Yes	1.3	0.0-3.3		–	–	
No	3.5	3.0-4.0		30.0	23.9-36.1	
HER2 status			0.522			0.170
Yes	3.0	2.0-4.0		–	–	
No	3.2	2.6-3.8		29.9	26.7-33.1	
Visceral metastasis			0.031			0.660
Yes	3.5	2.9-4.0		33.1	27.9-38.3	
No	2.8	2.7-2.9		27.9	0.0-64.6	
No. of previous therapy lines			0.121			0.504
< 3	2.8	2.5-3.1		–	–	
≥3	3.5	2.1-4.9		33.1	17.7-48.5	

After PSM, the baseline characteristics were relatively comparable. The PFS of the irinotecan group showed a longer trend of PFS without statistical significance at 3.2 months (95% CI 2.7–3.7) *vs* 2.1 months (95% CI 1.4–2.8), (P = 0.052) (**Figure 1**). Similarly, the OS of the irinotecan group was longer than the third-line survival without irinotecan, but it was not statistically significant at 33.1 months (95% CI 27.4–38.8) *vs* 18.0 months (95% CI 3.2–32.8), (P = 0.072) (**Figure 2**).

**Figure 3:**
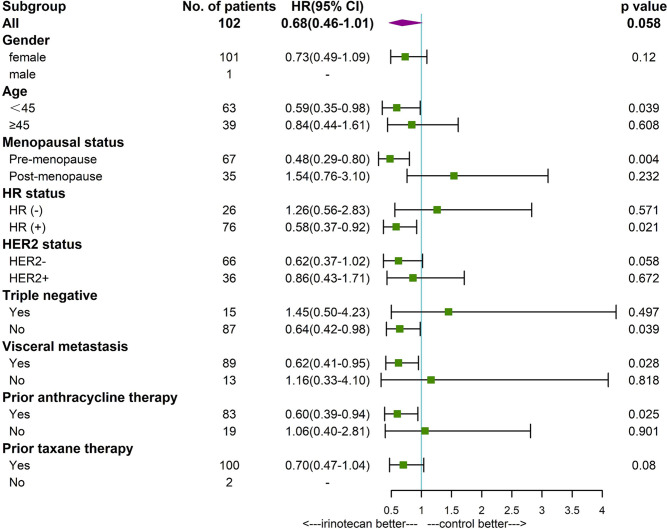
Forest plot of PFS by subgroup.

**Figure 4:**
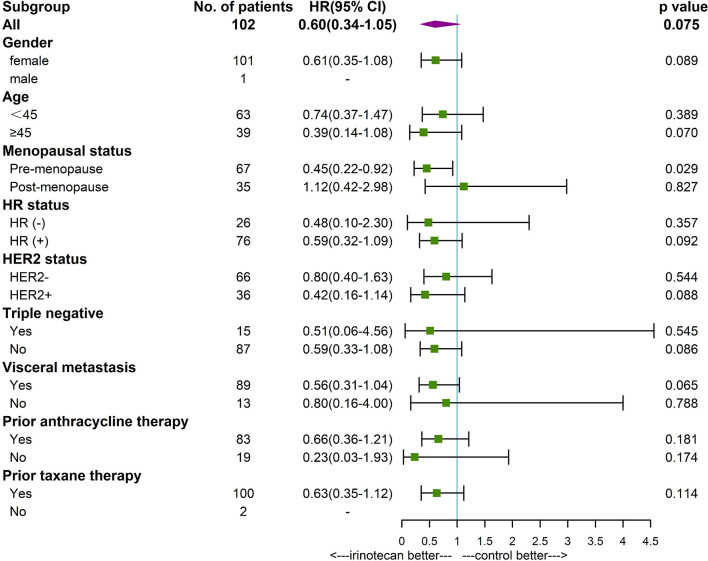
Forest plot of OS by subgroup.

Subgroup analysis found that patients younger than 45 years (P=0.039), premenopausal (P=0.004), HR positive (P=0.021), non-triple negative (P=0.039), with visceral metastases (P=0,028), and prior anthracycline therapy (P=0.025) had a longer PFS in patients treated with irinotecan. Premenopausal patients (P=0.029) with irinotecan had a longer OS. Other factors were not found to be significantly associated with patients’ PFS and OS (**Figures 3**, **4**).

### Subsequent Treatment

The large majority of patients received further therapy after irinotecan progression: 52 patients (86.7%) received systemic treatment (see specific treatment status in **Table 4**). As the higher proportion of patients receiving subsequent treatment would have inevitably affected OS, indicators such as the 2-year OS rate may more reliably reflect the efficacy of irinotecan in treating patients with MBC.

**Table 4:** Summary of subsequent line therapy.

Total cases	N = 51, %
Any subsequent treatment	46 (90.2)
Chemotherapy	43 (84.3)
Endocrine therapy	21 (41.2)
Anti-HER2 treatments	11(21.6)
Anti-angiogenesis treatments	6 (11.8)
Anti-PD-1 monoclonal antibody	2 (3.9)

## Discussion

This study retrospectively analyzed the efficacy of irinotecan in patients with MBC who had been heavily pretreated in the real world. Based on the limited available data in our database, we found that after failure of multi-line treatment of MBC patients, an irinotecan-containing regimen achieved an ORR of 15.7%, a DCR of 37.3%, and a median PFS of 3.2 months, achieving a median OS of 33.1 months.

The efficacy of irinotecan varies greatly among previous studies. A systemic analysis that enrolled 217 patients with refractory MBC in 5 irinotecan-based clinical studies confirmed a pooled RR of 48.8% (17). Other Phase I/II studies enrolled patients (n = 18–64) with MBC previously exposed to anthracycline and/or taxane-containing therapy using an irinotecan combination with other drugs such as cetuximab, temozolomide, docetaxel, gemcitabine, or etoposide. Those studies demonstrated an ORR of 5.6%–58.3%, a clinical benefit rate of 16%–97%, a median time to progression (TTP) of 1.4–14 months, and a median OS of 4.9–26 months (3, 18–27). There are few findings from large-scale, prospective, randomized studies on the use of irinotecan for MBC. We found only one Phase III randomized controlled trial comparing capecitabine with or without irinotecan in patients with MBC previously treated with anthracycline and taxane. The results suggest that for PFS, OS, and ORR, capecitabine plus irinotecan therapy is not significantly better than capecitabine. Until now, irinotecan’s position in breast cancer treatment regimens has not been established.

Compared to the current approved drugs for anthracycline and taxane-pretreated MBC such as capecitabine or eribulin, the PFS of our study was similar to those of eribulin or capecitabine, in which eribulin showed an ORR of 14.9%–20%, a clinical benefit rate of 30%, and a PFS of 3.9–4.0 months (28), while capecitabine’s ORR ranged from 14%–29% and exhibited a median TTP range from 3.1–5.9 months (29). After balancing age and molecular subtypes through PSM, the OS and PFS of MBC patients after the progression of anthracycline and paclitaxel with irinotecan may be better than those without irinotecan in third-line treatment, but it is not statistically significant. For patients with advanced MBC after failure of multi-line therapy, despite anthracycline and taxane having been used in the prior line, irinotecan may be considered as a treatment option when no better choice is available.

A handful of reports have suggested that irinotecan showed potentially promising results in triple negative breast cancer (3, 9), but unlike those studies, we found HR positive or non-triple negative patients had longer PFS treated with irinotecan compared with hormone receptor-negative patients. Second, we noticed that patients with younger than 45 years, premenopausal, with visceral metastasis, and prior anthracycline therapy had longer PFS. In particular, we observed a certain extension in PFS in patients with visceral metastasis with irinotecan (Previous treatment line of irinotecan was 4, indicating a possible drug-resistant population), suggesting that irinotecan is a posterior option for patients with visceral metastasis. Large-sample studies are needed to further identify patients with the highest likelihood of responding to treatment with irinotecan (13).

Given the dose-limiting toxicity of irinotecan and its inactivity in a large proportion of patients, it is more desirable to identify a biomarker to predict irinotecan’s activity. Some researchers have explored whether the increased topoisomerase 1 gene copy number or UGT1A1 polymorphisms can predict the response of the topoisomerase inhibitor irinotecan (3, 12). Due to the limited number of cases, no significant correlation has been found to be related to irinotecan’s response. Similarly, Cinzia Tesauro et al. investigated the relationship between CPT efficacy and TOP1 activity (including gene and protein levels) in BC cell lines (Luminal, HER2, and TNBC) *in vitro*, and found that TOP1 activity was not a marker for camptothecin sensitivity in breast cancer (9). Furthermore, researchers are also exploring several delivery strategies of SN-38, an active metabolite of irinotecan, showing a 100- to 1000-fold greater potency than irinotecan (30). Some preclinical work found liposomal irinotecan preferentially accumulates in metastatic lesions and acted as a reservoir for the release of irinotecan, improving anti-tumor activity with decreased toxicity in a number of animal models of human cancer (31, 32). And in a recent study(n=30), liposomal irinotecan showed favorable antitumor activity in heavily pretreated patients with or without brain metastasis, the reported objective response rate of 30%-34.5% with single drug and disease control rate 34.5%-50% (33).

Other evidence suggests that combining the topoisomerase I inhibitor deruxtecan with HER2-targeting antibody had excellent effects in breast cancer patients with HER2-positive and low-level HER2 expression. A Phase 2 study that enrolled 184 patients who received a median of 6 previous treatments followed by DS8201, a HER2-targeting antibody drug conjugate, found an RR of 60.9%, while the median duration of PFS was 16.4 months (34). From the above, we may see that it is possible to improve the efficacy of drugs by developing novel dosage formulations such as nanoparticles, liposomes, or pegylation; using drugs in combination with targeted agents; or using novel linker payload technology (compared with TDM1) (35).

### Limitations

This was a retrospective analysis of data collected at a single center. The sample size was limited. Several subtypes of breast cancers were mixed and irinotecan schedules were heterogenous.

## Conclusion

Irinotecan-containing regimens may achieve moderate objective response and showed a trend of survival benefit as a salvage treatment in MBC. The role of the topoisomerase 1 inhibitors in MBC still needs to be further validated in large-sample, prospective studies.

## Data Availability Statement

The raw data supporting the conclusions of this article will be made available by the authors, without undue reservation.

## Ethics Statement

The studies involving human participants were reviewed and approved by Clinical Test and Biomedical Ethics Committee of West China Hospital Sichuan University. Written informed consent for participation was not required for this study in accordance with the national legislation and the institutional requirements.

## Author Contributions

JS, XZ, PH, and TT collected data and performed statistical analysis. JS, XZ, and XY drafted the manuscript. HZ polished and checked the manuscript. TL conceived the study concept and design and confirmed the manuscript. All authors contributed to the article and approved the submitted version.

## Funding

This work was supported by the National Key Development Plan for Precision Medicine Research (grant number 2017YFC0910004), the 135 project for disciplines of excellence, West China Hospital, Sichuan University (grant number ZYGD18012), and Science and Technology Department of Sichuan Province project funding (2017FZ0062).

## Conflict of Interest

The authors declare that the research was conducted in the absence of any commercial or financial relationships that could be construed as a potential conflict of interest.

## Publisher’s Note

All claims expressed in this article are solely those of the authors and do not necessarily represent those of their affiliated organizations, or those of the publisher, the editors and the reviewers. Any product that may be evaluated in this article, or claim that may be made by its manufacturer, is not guaranteed or endorsed by the publisher.

## References

[1] Worldwide Cancer Statistics (WCRF). Available at: http://www.wcrf.org/cancer_sta.

[2] Winters S Martin C Murphy D Shokar NK. Breast Cancer Epidemiology, Prevention, and Screening. Prog Mol Biol Transl Sci (2017) 151:1–32. doi: 10.1016/bs.pmbts.2017.07.002 29096890

[3] Crozier JA Advani PP LaPlant B Hobday T Jaslowski AJ Moreno-Aspitia A. N0436 (Alliance): A Phase II Trial of Irinotecan With Cetuximab in Patients With Metastatic Breast Cancer Previously Exposed to Anthracycline and/or Taxane-Containing Therapy. Clin Breast Cancer (2016) 16(1):23–30. doi: 10.1016/j.clbc.2015.08.002 26381420 PMC4698217

[4] Fisusi FA Akala EO. Drug Combinations in Breast Cancer Therapy. Pharm Nanotechnol (2019) 7(1):3–23. doi: 10.2174/2211738507666190122111224 30666921 PMC6691849

[5] Park IH Im SA Jung KH Sohn JH Park YH Lee KS. Randomized Open Label Phase III Trial of Irinotecan Plus Capecitabine *Versus* Capecitabine Monotherapy in Patients With Metastatic Breast Cancer Previously Treated With Anthracycline and Taxane: PROCEED Trial (KCSG BR 11-01). Cancer Res Treat (2019) 51(1):43–52. doi: 10.4143/crt.2017.562 29458237 PMC6333992

[6] Cinieri S Chan A Altundag K Vandebroek A Tubiana-Mathieu N Barnadas A. Final Results of the Randomized Phase II NorCap-CA223 Trial Comparing First-Line All-Oral *Versus* Taxane-Based Chemotherapy for HER2-Negative Metastatic Breast Cancer. Clin Breast Cancer (2017) 17(2):91–9.e1. doi: 10.1016/j.clbc.2016.06.014 27756583

[7] Takashima T Mukai H Hara F Matsubara N Saito T Takano T. Taxanes *Versus* S-1 as the First-Line Chemotherapy for Metastatic Breast Cancer (SELECT BC): An Open-Label, Non-Inferiority, Randomised Phase 3 Trial. Lancet Oncol (2016) 17(1):90–8. doi: 10.1016/S1470-2045(15)00411-8 26617202

[8] Lorusso V Latorre A Giotta F. Chemotherapy Options Beyond the First Line in HER-Negative Metastatic Breast Cancer. J Oncol (2020) 2020:9645294. doi: 10.1155/2020/9645294 33312203 PMC7719522

[9] Tesauro C Simonsen AK Andersen MB Petersen KW Kristoffersen EL Algreen L. Topoisomerase I Activity and Sensitivity to Camptothecin in Breast Cancer-Derived Cells: A Comparative Study. BMC Cancer (2019) 19(1):1158. doi: 10.1186/s12885-019-6371-0 31783818 PMC6884793

[10] Basade M Mane A. Optimum Patient Selection for Irinotecan-Containing Regimens in Metastatic Colorectal Cancer: Literature Review and Lessons From Clinical Practice. Indian J Cancer (2021) 58(1):5–16. doi: 10.4103/ijc.IJC_507_19 33402591 10.4103/ijc.IJC_507_19

[11] Bailly C. Irinotecan: 25 Years of Cancer Treatment. Pharmacol Res (2019) 148:104398. doi: 10.1016/j.phrs.2019.104398 31415916

[12] Kümler I Balslev E Stenvang J Brünner N Ejlertsen B Jakobsen EH. Two Open-Label, Single Arm, non-Randomized Phase II Studies of Irinotecan for the Treatment of Metastatic Breast Cancer in Patients With Increased Copy Number of the Topoisomerase I Gene. BMC Cancer (2019) 19(1):573. doi: 10.1186/s12885-019-5788-9 31196001 PMC6567440

[13] Kumler I Brunner N Stenvang J Balslev E Nielsen DL. A Systematic Review on Topoisomerase 1 Inhibition in the Treatment of Metastatic Breast Cancer. Breast Cancer Res Treat (2013) 138(2):347–58. doi: 10.1007/s10549-013-2476-3 23512247

[14] Zhong X Luo T Deng L Liu P Hu K Lu D. Multidimensional Machine Learning Personalized Prognostic Model in an Early Invasive Breast Cancer Population-Based Cohort in China: Algorithm Validation Study. JMIR Med Inf (2020) 8(11):e19069. doi: 10.2196/19069 PMC7683252 33164899

[15] Perez EA Hillman DW Mailliard JA Ingle JN Ryan JM Fitch TR. Randomized Phase II Study of Two Irinotecan Schedules for Patients With Metastatic Breast Cancer Refractory to an Anthracycline, a Taxane, or Both. J Clin Oncol Off J Am Soc Clin Oncol (2004) 22(14):2849–55. doi: 10.1200/JCO.2004.10.047 15254052

[16] Peng Z Wei J Lu X Zheng H Zhong X Gao W. Diagnosis and Treatment Pattern Among Rural and Urban Breast Cancer Patients in Southwest China From 2005 to 2009. Oncotarget (2016) 7(47):78168–79. doi: 10.18632/oncotarget.11375 PMC5363653 27556301

[17] Lan H Li Y Lin CY. Irinotecan as a Palliative Therapy for Metastatic Breast Cancer Patients After Previous Chemotherapy. Asian Pac J Cancer Prev (2014) 15(24):10745–8. doi: 10.7314/apjcp.2014.15.24.10745 25605169

[18] Melisko ME Assefa M Hwang J DeLuca A Park JW Rugo HS. Phase II Study of Irinotecan and Temozolomide in Breast Cancer Patients With Progressing Central Nervous System Disease. Breast Cancer Res Treat (2019) 177(2):401–8. doi: 10.1007/s10549-019-05309-6 31172405

[19] Tan WW Hillman DW Salim M Northfelt DW Anderson DM Stella PJ. N0332 Phase 2 Trial of Weekly Irinotecan Hydrochloride and Docetaxel in Refractory Metastatic Breast Cancer: A North Central Cancer Treatment Group (NCCTG) Trial. Ann Oncol Off J Eur Soc Med Oncol (2010) 21(3):493–7. doi: 10.1093/annonc/mdp328 PMC5808723 19625343

[20] Anders C Deal AM Abramson V Liu MC Storniolo AM Carpenter JT. TBCRC 018: Phase II Study of Iniparib in Combination With Irinotecan to Treat Progressive Triple Negative Breast Cancer Brain Metastases. Breast Cancer Res Treat (2014) 146(3):557–66. doi: 10.1007/s10549-014-3039-y PMC4112043 25001612

[21] Hayashi H Tsurutani J Satoh T Masuda N Okamoto W Morinaga R. Phase II Study of Bi-Weekly Irinotecan for Patients With Previously Treated HER2-Negative Metastatic Breast Cancer: KMBOG0610B. Breast Cancer (Tokyo Japan) (2013) 20(2):131–6. doi: 10.1007/s12282-011-0316-z 22124996

[22] Segar JM Reed D Stopeck A Livingston RB Chalasani P. A Phase II Study of Irinotecan and Etoposide as Treatment for Refractory Metastatic Breast Cancer. Oncol (2019) 24(12):1512–e267. doi: 10.1634/theoncologist.2019-0516 PMC6975935 31383812

[23] Tanaka T Tanaka M Furusawa H Kamada Y Sagara Y Anan K. Pilot Study of Irinotecan and S-1 (IRIS) for Advanced and Metastatic Breast Cancer. Anticancer Res (2020) 40(8):4779–85. doi: 10.21873/anticanres.14480 32727805

[24] Moulder S Valkov N Neuger A Choi J Lee JH Minton S. Phase 2 Study of Gemcitabine and Irinotecan in Metastatic Breast Cancer With Correlatives to Determine Topoisomerase I Localization as a Predictor of Response. Cancer (2008) 113(10):2646–54. doi: 10.1002/cncr.23916 18823053

[25] Stathopoulos GP Tsavdaridis D Malamos NA Rigatos SK Kosmas C Pergantas N. Irinotecan Combined With Docetaxel in Pre-Treated Metastatic Breast Cancer Patients: A Phase II Study. Cancer Chemother Pharmacol (2005) 56(5):487–91. doi: 10.1007/s00280-005-1006-3 15868147

[26] Otsuka H Fujii T Toh U Iwakuma N Takahashi R Mishima M. Phase II Clinical Trial of Metronomic Chemotherapy With Combined Irinotecan and Tegafur-Gimeracil-Oteracil Potassium in Metastatic and Recurrent Breast Cancer. Breast Cancer (Tokyo Japan) (2015) 22(4):335–42. doi: 10.1007/s12282-013-0483-1 23827973

[27] Lee KS Park IH Nam BH Ro J. Phase II Study of Irinotecan Plus Capecitabine in Anthracycline- and Taxane- Pretreated Patients With Metastatic Breast Cancer. Investigat New Drugs (2013) 31(1):152–9. doi: 10.1007/s10637-012-9824-8 22562702

[28] Perez-Garcia JM Cortes J. The Safety of Eribulin for the Treatment of Metastatic Breast Cancer. Expert Opin Drug Saf (2019) 18(5):347–55. doi: 10.1080/14740338.2019.1608946 31107111

[29] Chan A Verrill M. Capecitabine and Vinorelbine in Metastatic Breast Cancer. Eur J Cancer (2009) 45(13):2253–65. doi: 10.1016/j.ejca.2009.04.031 19464166

[30] Lin HC Chuang CH Cheng MH Lin YC Fang YP. High Potency of SN-38-Loaded Bovine Serum Albumin Nanoparticles Against Triple-Negative Breast Cancer. Pharmaceutics (2019) 11(11):569. doi: 10.3390/pharmaceutics11110569 PMC6920977 31683822

[31] Mohammad AS Griffith JI Adkins CE Shah N Sechrest E Dolan EL. Liposomal Irinotecan Accumulates in Metastatic Lesions, Crosses the Blood-Tumor Barrier (BTB), and Prolongs Survival in an Experimental Model of Brain Metastases of Triple Negative Breast Cancer. Pharm Res (2018) 35(2):31. doi: 10.1007/s11095-017-2278-0 29368289 PMC5884086

[32] Bernards N Ventura M Fricke IB Hendriks BS Fitzgerald J Lee H. Liposomal Irinotecan Achieves Significant Survival and Tumor Burden Control in a Triple Negative Breast Cancer Model of Spontaneous Metastasis. Mol Pharm (2018) 15(9):4132–8. doi: 10.1021/acs.molpharmaceut.8b00540 30059232

[33] Sachdev JC Munster P Northfelt DW Han HS Ma C Maxwell F. Phase I Study of Liposomal Irinotecan in Patients With Metastatic Breast Cancer: Findings From the Expansion Phase. Breast Cancer Res Treat (2021) 185(3):759–71. doi: 10.1007/s10549-020-05995-7 PMC7921078 33201358

[34] Modi S Saura C Yamashita T Park YH Kim SB Tamura K. Trastuzumab Deruxtecan in Previously Treated HER2-Positive Breast Cancer. N Engl J Med (2020) 382(7):610–21. doi: 10.1056/NEJMoa1914510 PMC7458671 31825192

[35] Ocana A Amir E Pandiella A. HER2 Heterogeneity and Resistance to Anti-HER2 Antibody-Drug Conjugates. Breast Cancer Res (2020) 22(1):1. doi: 10.1186/s13058-020-1252-7 PMC6995165 32005279

